# The Dipole Potential Modifies the Clustering and Ligand Binding Affinity of ErbB Proteins and Their Signaling Efficiency

**DOI:** 10.1038/srep35850

**Published:** 2016-10-24

**Authors:** Tamás Kovács, Gyula Batta, Tímea Hajdu, Ágnes Szabó, Tímea Váradi, Florina Zákány, István Csomós, János Szöllősi, Peter Nagy

**Affiliations:** 1 Department of Biophysics and Cell Biology, Faculty of Medicine, University of Debrecen, Egyetem square 1, 4032 Debrecen, Hungary; 2 MTA-DE Cell Biology and Signaling Research Group, Faculty of Medicine, University of Debrecen, Egyetem square 1, 4032 Hungary

## Abstract

Although activation of the ErbB family of receptor tyrosine kinases (ErbB1-4) is driven by oligomerization mediated by intermolecular interactions between the extracellular, the kinase and the transmembrane domains, the transmembrane domain has been largely neglected in this regard. The largest contributor to the intramembrane electric field, the dipole potential, alters the conformation of transmembrane peptides, but its effect on ErbB proteins is unknown. Here, we show by Förster resonance energy transfer (FRET) and number and brightness (N&B) experiments that the epidermal growth factor (EGF)-induced increase in the homoassociation of ErbB1 and ErbB2 and their heteroassociation are augmented by increasing the dipole potential. These effects were even more pronounced for ErbB2 harboring an activating Val → Glu mutation in the transmembrane domain (NeuT). The signaling capacity of ErbB1 and ErbB2 was also correlated with the dipole potential. Since the dipole potential decreased the affinity of EGF to ErbB1, the augmented growth factor-induced effects at an elevated dipole potential were actually induced at lower receptor occupancy. We conclude that the dipole potential plays a permissive role in the clustering of ErbB receptors and that the effects of lipid rafts on ligand binding and receptor signaling can be partially attributed to the dipole potential.

ErbB proteins are known to a wide array of scientists ranging from cell biologists to clinical researchers^1^,^2^. The interest in this family of receptor tyrosine kinases stems from the fact that its members, ErbB1-4 (also known as HER1-4), are prototypes of dimerization-activated proteins, and they are involved in the pathogenesis of cancer^3^. Activated ErbB proteins turn on signaling via the MAPK and PI3K (phosphatidylinositol 3-kinase) pathways leading to proliferation, increased motility or differentiation depending on the context of the stimulation^2^. The fact that ErbB proteins are drugable targets led to the development of targeted cancer therapeutics^4^.

Clustering plays a key role in the activation of ErbB proteins. The dogma, formulated for ErbB1 (also known as epidermal growth factor (EGF) receptor), involves the ligand-induced dimerization of monomeric receptors as a result of a transition of the extracellular domain from a closed to an extended conformation in which the dimerization arm is exposed^5^. Several findings suggest that a process more complex than the one outlined above is involved in receptor activation. Binding of a single EGF molecule leads to the formation of an asymmetric dimer believed to form the structural basis of negative cooperativity in ligand binding^6^,^7^. Preformed ErbB1 dimers are also thought to exist^8^, although mainly at non-physiologically high expression levels^9^. Quantitative live cell imaging revealed that unliganded ErbB1 receptors form transient dimers^10^. Higher order clusters have also been identified for ErbB1-3^8^,^11^,^12^.

In addition to the extracellular domain the transmembrane and the intracellular domains also play a role in the clustering of ErbB receptors. The structure of the autoinhibited conformation of the ErbB1 kinase domain resembles that of inactive cyclin-dependent kinases. Upon activation two kinase domains form an asymmetric dimer in which the “receiver” kinase is turned on by the “activator” kinase^13^. A similar sharing of tasks has also been indirectly shown for the kinase domains in ErbB heterodimers^14^.

We have a much less clear view about the role of the transmembrane domain (TMD) in dimerization. Although the linkage between the extra- and intracellular domains provided by the transmembrane and juxtamembrane domains seems to be very flexible^15^,^16^, several lines of evidence suggest that the TMD does have a remarkable role in receptor activation. A Val→Glu point mutation in the TMD of human ErbB2 (NeuT) or its rat homolog increases the formation of active ErbB2 homodimers^17^,^18^. (Although the designation Neu was originally used to describe an oncogene from nitrosoethylurea-induced rat neuroblastomas, it is nowadays widely used to refer to the human ErbB2/HER2 oncoprotein as well. All the protein or gene symbols in the paper refer to the human versions). Replacement of the same valine with isoleucine as a result of single nucleotide polymorphism leads to reduced risk of breast cancer^19^. It is believed that two terminal GXXXG motifs stabilize two different TMD dimers^17^. The two motifs are believed to differ regarding their role in stabilizing homo- and heterodimeric interactions between TMDs^20^. Homodimers of the TMDs of ErbB1 and ErbB2 and their heterodimers show the strongest tendency for association among the possible combinations of ErbB proteins^21^. The involvement of the TMD in receptor activation can be exploited for therapeutic purposes by oligopeptide sequences competitively blocking receptor dimerization, activation and tumor growth^22^. A global model for the activation of ErbB1 suggests that the TMD and the kinase domains have intrinsic dimerization and self-activation tendency, which is counteracted by the tethered conformation of the extracellular domain, by the formation of inactive kinase dimers and by interactions of the juxtamembrane segment with anionic lipids of the membrane^23^,^24^,^25^,^26^,^27^. This inhibitory mechanism is relieved by ligand binding to the extracellular domain^27^.

The electric field in the membrane alters the conformation of the TMD^28^. Besides the transmembrane potential, whose magnitude is 50–100 mV, the dipole potential must also affect the TMD since its magnitude is several hundred millivolts, and due to the steeper change in the potential the electric field generated by the dipole potential is 10^8^–10^9^ V/m as opposed to the much weaker (~2.5·10^7^ V/m) electric field associated with the transmembrane potential^28^. The dipole potential is generated by the dipole moments of lipid carbonyl groups, cholesterol and the non-random orientation of water molecules on the surface of the membrane^29^,^30^. The dipole potential has been shown to alter transmembrane diffusion of charged molecules^28^, ligand binding^31^ and the structure and function of ion pumps^32^. Owing to the richness of lipid rafts in cholesterol the dipole potential is assumed to be larger in these microdomains than in the rest of the membrane^33^.

Although the dipole potential is expected to influence the conformation of the TMD of membrane proteins, its effect has not been tested on ErbB proteins. Here, we show that alterations in the dipole potential modify the clustering and signaling properties of ErbB1 and ErbB2, and conclude that the dipole potential plays a permissive role in the activation of these receptors.

## Results

### Binding of EGF is Significantly Inhibited by an Elevated Dipole Potential

**Figure 1:**
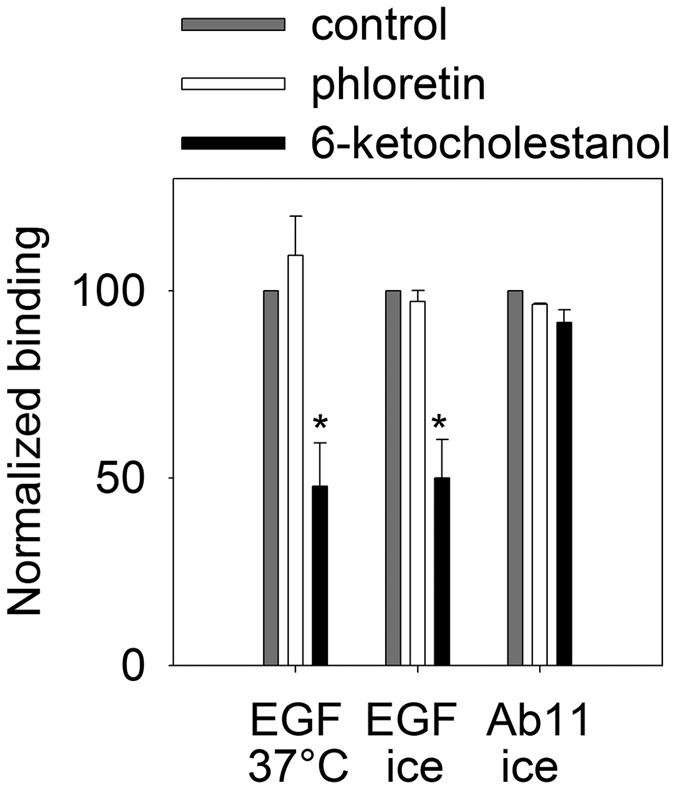
The effect of the dipole potential on the cell surface expression and ligand binding of ErbB1. Starved SKBR-3 cells were treated with phloretin or 6-ketocholestanol in the presence of Pluronic F127 followed by incubating them with 100 ng/ml (~16 nM) tetramethylrhodamine-conjugated EGF at 37 °C for 5 min, on ice for 30 min or with AlexaFluor546-Ab11 against ErbB1 on ice for 30 min. Fluorescence intensities were background-corrected and normalized to the intensity of control samples treated with Pluronic F127 only. The mean of three independent flow cytometric measurements (±standard error of the mean) are shown in the figures. Asterisks indicate significant differences compared to the control samples (p < 0.05, ANOVA followed by Tukey’s HSD test).

Phloretin and 6-ketocholestanol were used to decrease and increase the dipole potential, respectively (see Supplementary Results and Fig. S1 for details of ratiometric measurement of the dipole potential). First, the binding affinity of fluorescent EGF to three different cell lines was determined and the analysis revealed that an elevated dipole potential significantly reduced the affinity of EGF to ErbB1 (Table 1, Fig. S2). Incorporation of a fluorescent dye may alter the interaction of EGF with the dipole potential. In order to exclude the possibility that the observed inhibited binding of EGF at a high dipole potential was due to the presence of the fluorescent label, competitive binding experiments were carried out. The binding affinity of unlabeled EGF was reduced by a high dipole potential to a similar extent as for fluorescent EGF (Table 1, Fig. S2). Next, the binding of fluorescent EGF was tested under conditions used for stimulation experiments. The binding of fluorescent EGF was reduced by ~50% at an elevated dipole potential when growth factor labeling was performed either on ice or at 37 °C (Fig. 1 showing representative results for SKBR-3). These results are in agreement with predictions based on the binding curves. While the binding of EGF was significantly modified by the dipole potential, the expression of the receptor, ErbB1, was not modified as shown by the unaltered binding of an ErbB1-specific antibody (Fig. 1). Binding of EGF was restricted to the cell membrane in the experiment carried out on ice, while the growth factor was endocytosed and reached intracellular compartments at 37 °C. These experiments convincingly showed that the ligand binding ability of ErbB1 was significantly inhibited by an elevated dipole potential.

### The Effect of the Dipole Potential on the Clustering of ErbB1 and ErbB2

**Figure 2:**
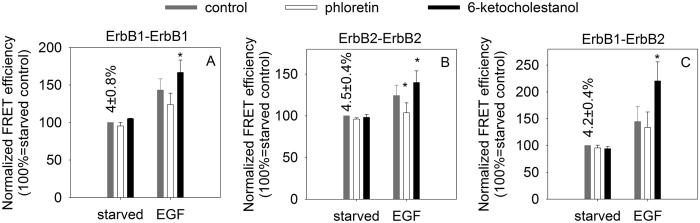
The effect of the dipole potential on the homo- and heteroassociations of ErbB1 and ErbB2. ErbB1 homoassociation (**A**), ErbB2 homoassociation (**B**), and the heteroassociation of ErbB1 and ErbB2 (**C**) in control, phloretin- and 6-ketocholestanol-treated cells were measured by flow cytometric FRET. In each measurement, the calculated mean FRET efficiencies were normalized to the mean FRET efficiencies observed in samples treated with Pluronic F127. The numbers above the columns represent the unnormalized mean FRET efficiency (±standard error of the mean) of the serum-starved control sample. The columns and the error bars represent the means and their standard errors, respectively, determined from four independent measurements. Two-way ANOVA was performed and pairwise comparisons were carried out by Tukey’s HSD test. Asterisks indicate significant differences compared to the control sample of the respective treatment group (p < 0.05, ANOVA followed by Tukey’s HSD test). Representative FRET histograms used for calculating data presented in this figure are shown in Fig. S3.

**Figure 3:**
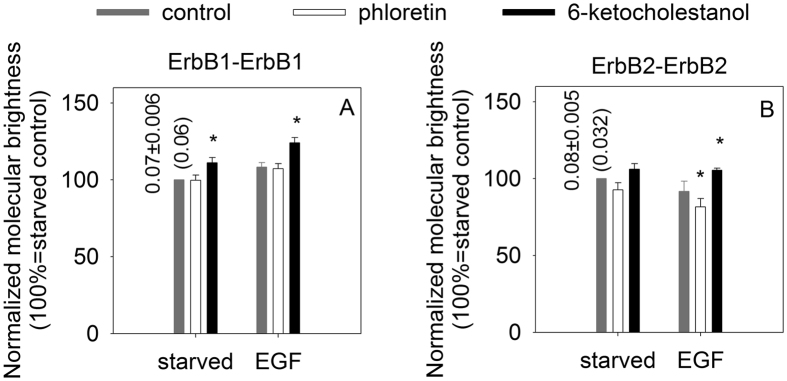
The effect of the dipole potential on the homoassociation of ErbB1 and ErbB2 measured by N&B analysis. The homoassociation of ErbB1 (**A**) and ErbB2 (**B**) were determined in F1-4 cells stably expressing ErbB1-EGFP and HeLa cells transfected with ErbB2-mYFP, respectively. The observed molecular brightness was normalized to the mean molecular brightness of the starved sample treated with Pluronic F127. The normalized molecular brightness values of ErbB1-EGFP and ErbB2-mYFP (mean ± standard error of the mean), determined from six independent experiments, are shown in the graph. The numbers above the columns represent the unnormalized molecular brightness (±standard error of the mean) of the serum-starved control sample. The values in parentheses are the molecular brightness values of monomeric fluorophores. Asterisks indicate a significant difference compared to the control sample of the respective treatment group (p < 0.05, ANOVA followed by Tukey’s HSD test). Representative brightness histograms used for calculating data presented in this figure are shown in Fig. S3.

**Figure 4:**
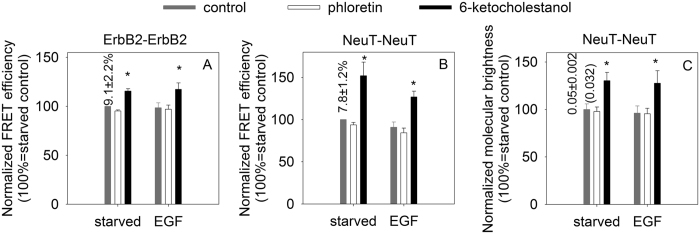
The effect of the dipole potential on the homoclustering of NeuT. HeLa cells were transfected with wild-type ErbB2 (**A**), with NeuT (**B**) or with NeuT fused with mYFP (**C**) and their homoclustering was measured by flow cytometric FRET (**A,B**) or N&B analysis (**C**). The calculated molecular brightness and FRET values were normalized to the mean of the starved sample treated with Pluronic F127. The normalized molecular brightness or FRET values (mean ± standard error of the mean), determined from six independent experiments, are shown in the graph. The numbers above the columns on the left represent the unnormalized molecular brightness or FRET values (±standard error of the mean) of the serum-starved control sample. The value in parentheses is the molecular brightness value of a monomeric fluorophore. Asterisks indicate a significant difference compared to the control sample of the respective treatment group (p < 0.05, ANOVA followed by Tukey’s HSD test).

Since transmembrane signaling induced by EGF is preceded by clustering of ErbB receptors, we investigated whether alterations in the dipole potential have any effect on receptor oligomerization in quiescent or stimulated cells. According to flow cytometric FRET (Figs 2 and S3) and N&B (Figs 3 and S3) experiments the dipole potential exerted only minor effects on the homoassociation of ErbB1 and ErbB2 in non-stimulated cells with an increased homoclustering of ErbB1 at an elevated dipole potential being the only significant effect in N&B measurements. In accordance with previous results^9^ ErbB1 was mainly monomeric in unstimulated cells, since the molecular brightness of ErbB1-EGFP (0.07) was comparable to that of monomeric EGFP (0.06). On the other hand, ErbB2 formed clusters in quiescent cells judged from the almost threefold difference between the molecular brightness of ErbB2-mYFP (0.08) and that of monomeric mYFP (0.032). Homoclustering of the TMD mutant NeuT was significantly enhanced even in unstimulated cells by an elevated dipole potential according to both FRET and N&B measurements (Fig. 4B,C). Although the homoclustering of transfected wild-type ErbB2 was also slightly increased by the dipole potential according to FRET measurements, this effect was less significant than that on NeuT (15% and 52% 6-ketocholestanol-induced relative increase in the FRET efficiency for wild-type ErbB2 and NeuT, respectively; p < 0.05).

In order to reveal the effect of the dipole potential on EGF-induced receptor clustering both the FRET and the molecular brightness values had to be normalized in order to eliminate variability in the values in unstimulated cells. Contrary to resting cells, an elevated dipole potential significantly and systematically promoted the EGF-induced homoassociation of ErbB1 and ErbB2 according to FRET and N&B measurements (Figs 2A,B and 3). Although the phloretin-induced decrease in the dipole potential had a less predictable effect, the EGF-induced homoclustering of ErbB2 was significantly inhibited by phloretin (Figs 2B and 3B). The opposite behavior of ErbB1 and ErbB2 homoclusters in N&B measurements after EGF stimulation is in accordance with our previous results in which we showed that EGF induces ErbB1-2 heteroassociations by recruiting ErbB2 from large-scale ErbB2 homoclusters thereby decreasing the size of ErbB2 homo-oligomers^9^. An elevated dipole potential exerted an approximately 2-times larger effect on the EGF-induced homoclustering of NeuT as compared to that on wild-type ErbB2 according to FRET and N&B measurements since the relative, 6-ketocholestanol-induced change in the FRET values in EGF-stimulated samples were 19% and 40% for wild-type ErbB2 and NeuT, respectively, and the 6-ketocholestanol-induced rise in the molecular brightness values were 13% and 33% under the same two conditions (Figs 3–4, p<0.05).

Since ErbB2 is the preferred heterodimerization partner of EGF-activated ErbB1^34^, the investigation of the effect of the dipole potential on clusters of ErbB1 and ErbB2 would be incomplete without measuring their heteroassociation. According to flow cytometric FRET measurements the dipole potential did not have any effect on the heteroclustering of ErbB1 with ErbB2 in quiescent cells, while the EGF-induced increase in the interaction of the two receptors was significantly higher in cells in which the dipole potential was elevated by 6-ketocholestanol (Fig. 2C). We concluded that the homo- and heteroassociations of ErbB1 and ErbB2, especially in ligand-stimulated cells, show a positive correlation with the dipole potential.

### Dipole potential-induced changes in EGF-induced signaling

**Figure 5:**
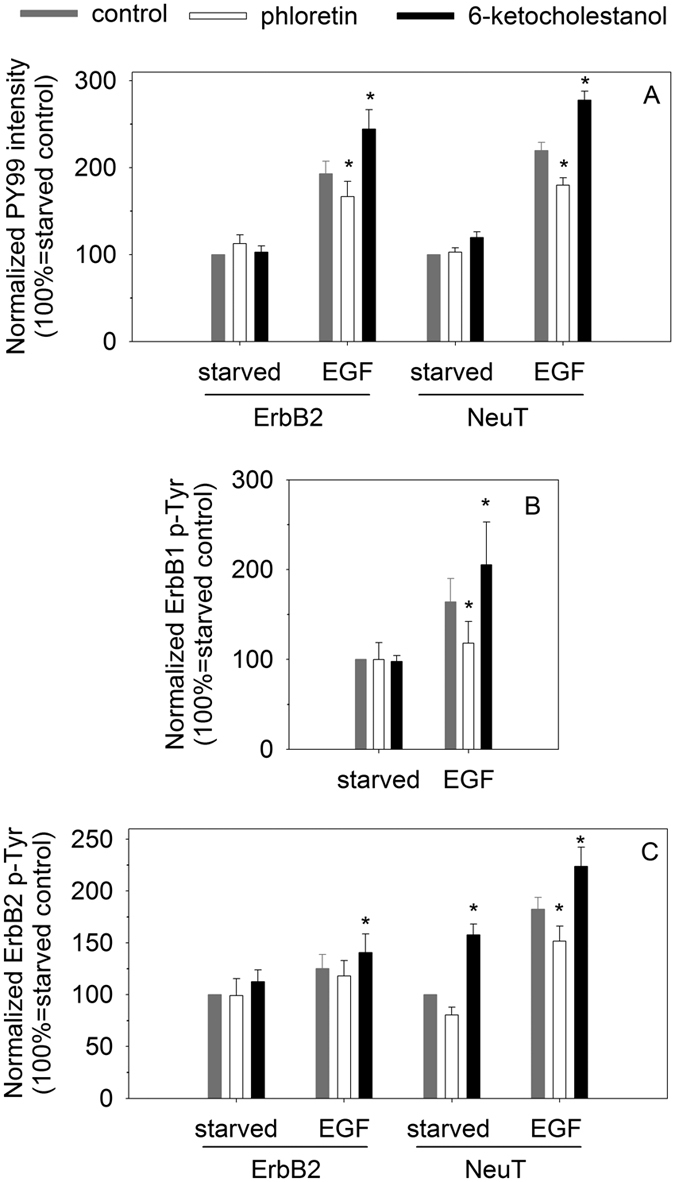
The effect of the dipole potential on EGF-induced tyrosine phosphorylation. General tyrosine phosphorylation was determined by PY99 (**A**), whereas ErbB1 phosphorylation at Tyr1068 and ErbB2 phosphorylation at Tyr1248 were measured by anti-pEGFR (**B**), and Ab18 antibodies (**C**), respectively. The mean fluorescence intensities of control and EGF-treated samples labeled with AlexaFluor546-goat-anti-mouse antibodies were normalized to the means of samples treated with Pluronic F127. The means (±standard error of the mean) of four independent measurements are shown. Two-way ANOVA was performed and pairwise comparisons were carried out by Tukey’s HSD test. Asterisks indicate significant differences compared to the control sample of the respective treatment group (p < 0.05).

Ligand-induced clustering of ErbB receptors leads to phosphorylation of tyrosine residues in the receptors and spreading of the tyrosine phosphorylation signal. In order to test whether the dipole potential-related changes observed in the clustering of ErbB1 and ErbB2 are mirrored in their signaling the level of tyrosine phosphorylation was determined in quiescent and EGF-stimulated cells using antibodies against phosphotyrosine, ErbB1 phosphorylated at Tyr1068 and ErbB2 phosphorylated at Tyr1248. Similar to the FRET and N&B experiments normalization of the tyrosine phosphorylation to unstimulated control cells eliminated the variability in the baseline level of activation. Alteration of the dipole potential did not modify the low level of tyrosine phosphorylation in unstimulated cells, while the EGF-induced response was correlated with the dipole potential (Fig. 5). Similar to the FRET and N&B measurements the effect of the dipole potential on NeuT was more pronounced than on wild-type ErbB2. We concluded that the growth factor-induced tyrosine phosphorylation response of cells was also significantly modified by the dipole potential.

### Correlation between the activation of ErbB2, its raft localization and the dipole potential

**Figure 6:**
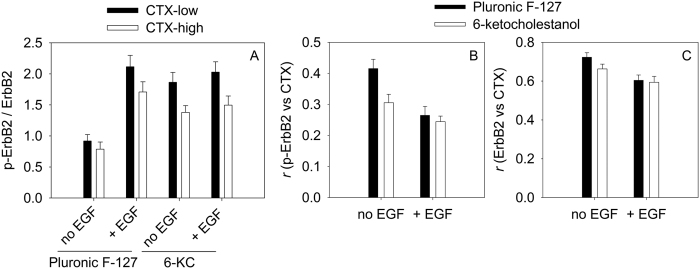
Correlation between the activation of ErbB2, its raft localization and the dipole potential. SKBR-3 cells were pretreated with 6-ketocholestanol (6-KC) to increase the dipole potential. Control cells were only incubated with Pluronic F-127. Cells were stimulated with EGF followed by staining all membrane-localized ErbB2 with trastuzumab, tyrosine phosphorylated ErbB2 (p-ErbB2) with Ab18 and GM1 gangliosides, localized in lipid rafts, with subunit B of cholera toxin (CTX). The tyrosine phosphorylation of ErbB2 normalized to its membrane expression (p-ErbB2/ErbB2) was separately evaluated for image regions showing high and low CTX labeling (**A**). The Pearson correlation coefficient (*r*) between p-ErbB2 and CTX (**B**) and between total membrane ErbB2 and CTX (**C**) were calculated. Error bars represent the standard error of the mean determined from 25 images recorded in three independent experiments.

Since the dipole potential is assumed to be different in lipid rafts than in the rest of the membrane, we expected some sort of correlation between the activation state of ErbB2, its raft localization and the dipole potential. Cells treated with 6-ketocholestanol to increase their dipole potential were compared to control cells. The tyrosine phosphorylation of ErbB2 was normalized to its expression level and separately evaluated in raft and non-raft regions of the cell membrane corresponding to areas with high and low CTX labeling, respectively. In cells with an unmodified dipole potential there was no difference between the normalized tyrosine phosphorylation of ErbB2 inside and outside lipid rafts (Fig. 6A). Increasing the dipole potential enhanced the activation state of ErbB2 and it did so more pronouncedly outside lipid raft regions. EGF stimulation led to ErbB2 tyrosine phosphorylation both inside and outside lipid rafts in cells with an unmodified dipole potential and the increase was more pronounced in regions outside lipid rafts. EGF stimulation in cells with an increased dipole potential did not increase the normalized tyrosine phosphorylation of ErbB2 further. Differences between the effects of EGF stimulation presented in Figs 5 and 6 may be related to the fact that trypsinized cells were stimulated in flow cytometric experiments (Fig. 5) while attached cells were investigated in the measurements presented in Fig. 6, and trypsinization alone can lead to stimulation of signaling pathways and diminish growth factor-induced effects (Fig. S4). The results suggest that 6-ketocholestanol-induced increase in the dipole potential affects ErbB2 tyrosine phosphorylation more significantly outside lipid rafts. The fact that EGF stimulation led to a larger rise in ErbB2 tyrosine phosphorylation outside lipid rafts suggests that the growth factor either preferentially activates ErbB2 outside lipid rafts or activated ErbB2 migrates out of lipid rafts. This assumption was corroborated by a significant decrease in the correlation coefficient between phosphorylated ErbB2 and CTX staining upon EGF and 6-ketocholestanol treatments (Fig. 6B) while the effects on the correlation between ErbB2 and CTX intensities were less substantial. In conclusion, the results suggest that activation of ErbB2 by either EGF or increased dipole potential preferentially takes place outside lipid rafts.

## Discussion

In the current manuscript we showed that the dipole potential is significantly correlated with the clustering of ErbB1 and ErbB2. The effect was systematically observed in EGF-stimulated cells, whereas changes in the dipole potential did not exert substantial effects on ErbB1 and ErbB2 in unstimulated cells. The increased growth factor-induced association of ErbB proteins at an elevated dipole potential was correlated with enhanced signaling which is in line with receptor dimers or clusters being the active signaling units. However, the fact that increased signaling at an elevated dipole potential was correlated with reduction of ligand binding was unexpected. If this suppressed ligand binding at an elevated dipole potential is taken into account, the dipole potential-induced augmentation of the EGF effect is even larger since a lower amount of bound ligand brings about the larger changes in the clustering and phosphorylation of ErbB1 and ErbB2. This apparent contradiction can be resolved by assuming that the increase of the dipole potential favors the active conformation of the transmembrane domain dimers which eventually induces conformational transitions in the extra- and intracellular domains as well resembling or identical to those induced by growth factor binding. In light of the above reasoning a lower amount of bound ligand at an elevated dipole potential can induce stronger signaling since both growth factor binding and the dipole potential induce the conformational changes leading to receptor activation.

The reduced affinity of EGF binding is also in line with the dipole potential-induced conformational change in the extracellular domain. It has been shown that the ligand binding site is structurally restrained in ErbB1 dimers^6^,^7^. Consequently, ligand independent, dipole potential-driven formation of receptor dimers leads to generation of compressed ligand binding pockets and reduced affinity.

Lipid rafts regulate EGF-mediated signaling since both ErbB1^35^ and ErbB2^36^,^37^ have been shown to be raft resident. Lipid rafts are presumed to play a bipartite role in the clustering and signaling of ErbB proteins. They inhibit ligand binding^38^,^39^,^40^, but potentiate growth factor-induced signaling at the same time^35^,^36^,^37^. Since the dipole potential is stronger in lipid rafts than in non-raft domains^33^, it must not be overlooked when interpreting raft-dependent effects on receptor function. Indeed, our results show that the dipole potential inhibits ligand binding and potentiates EGF-mediated signaling which exactly mirrors the bipartite effect of lipid rafts. The magnitude of increase in the dipole potential induced by 6-ketocholestanol treatment must be higher in non-raft domains since the dipole potential-induced activation of ErbB2 was more pronounced outside lipid rafts.

We considered six possible models for explaining the phenomenon that the dipole potential only affects ligand-induced clusters without influencing constitutive ones.

1. The dipole potential may only exert a permissive effect on clustering which is not strong enough for altering the weak and transient constitutive dimers and oligomers.
2. It has been suggested that constitutive dimers or oligomers are generated by corralling or confinement by the cytoskeleton^10^, while actively signaling receptor clusters are held together by protein-protein interactions^41^,^42^. Since it is unlikely that the membrane dipole potential affects corralling and confinement, it only exerts effects on ligand-induced clusters.
3. Parallel alpha helices, present in dimers of single-pass transmembrane proteins, exert a repulsive electrostatic force on each other^43^. The N-terminal and C-terminal dimerization motifs are thought to contribute to the stabilization of dimers of the TMD with the N-terminal one implicated in stabilizing active dimers^17^,^24^,^27^. Although a lot of controversy surrounds the magnitude of the “macroscopic” net dipole moment equivalent to the α helical TMD^44^, it is reasonable to approximate it by placing half unit negative and positive charges near the C- and N-terminus, respectively^45^. According to a semiquantitative model presented in Fig. S5 the magnitude of the membrane dipole potential is similar to the electric potential leading to the repulsion of the helix termini. The membrane dipole potential counterbalances this repulsion at the N-termini leading to the stabilization of interaction between the N-terminal dimerization motifs thought to be responsible for active dimers. On the other hand, the membrane dipole potential does not have such an effect on the C-terminal dimerization motifs. Since the dimerization motifs have been shown to interact specifically with each other, the dipole potential only alters the strength of their interaction. The larger effect of the dipole potential on NeuT as compared to wild-type ErbB2 is in agreement with the above reasoning since the Val→Glu mutation favors interactions between the N-terminal dimerization motifs stabilizing active dimers^17^.
4. Interactions of the juxtamembrane segment with negatively charged lipids in the inner leaflet of the plasma membrane have been shown to prevent the formation of active receptor dimers, while dimerization of the juxtamembrane domains orient the kinase domain to adopt their active conformation^23^,^24^,^25^. Since the juxtamembrane domain is embedded in the hydrophobic core of the membrane^24^, the positively charged lysine residues in the JM-A segment may be repelled by the positive lobe of the dipole potential. In this way, an increased dipole potential may destabilize the membrane embedding of the JM-A segment favoring the formation of juxtamembrane domain dimers in which the positively charged lysine residues do not penetrate the membrane.
5. Exposure of the TMD helix termini to water decreases their dipole moment and repulsion^44^. Consequently, if the dipole potential decreased membrane thickness, less repulsion between TMDs would favor receptor clustering. However, it has been shown that neither phloretin, nor 6-ketocholestanol changes the membrane thickness arguing against this model^46^,^47^.
6. Since the interaction with lipids affects the conformation of ErbB proteins, changes in the dipole potential and consequent alterations in lipid-protein interactions may change the distribution of ErbB receptors between raft and non-raft domains. The majority of studies found that lipid rafts inhibit ligand binding and signaling mediated by ErbB1 and that ErbB1 activation takes place upon concomitant migration of ErbB1 out of lipid rafts^38^,^48^, although alternative views have also been presented^35^. Our results imply that activation of ErbB2 by EGF preferentially takes place outside lipid raft domains and an increased dipole potential mimics this effect. Therefore, dipole potential-induced redistribution of receptors can also enhance their association and signaling.

By one of these mechanisms monomers of ErbB proteins are more prone to dimerization after 6-ketocholestanol treatment. In this way, more monomers are available to form homo- and heterodimers after growth factor stimulation. Therefore, EGF-induced formation of ErbB1-2 heterodimers does not lead to decreased homoassociation of ErbB1 or ErbB2 because the dimers are formed from the dimerization-prone pool of monomers.

The dipole potential may also have implications for the evolution and treatment of cancer. Tumor cells are characterized by an increased density of cholesterol-rich lipid rafts^49^. Since the dipole potential is higher in lipid rafts than in the rest of the membrane^33^, this phenomenon may activate receptor tyrosine kinases in cancer cells. 2-hydroxylated unsaturated fatty acids have been shown to decrease the dipole potential and to have beneficial effects in the treatment of cancer and inflammatory diseases implying that attenuation of signaling upon lowering the dipole potential may even have therapeutic implications^50^.

In summary, we have shown that the dipole potential exerts significant effects on the ligand binding, clustering and signaling of ErbB proteins. We suggest that the dipole potential primarily affects receptor dimerization mediated by TMDs. This dimerization induced by an elevated dipole potential will bring about TMD-driven receptor activation leading to reduced affinity of growth factor binding and increased signaling mediated by dimers stabilized by interactions among the extracellular domains and the TMD dimerization motifs stabilizing active dimers^17^. Our observations emphasize that the dipole potential must not be overlooked when interpreting the effect of the membrane environment on receptor clustering.

## Materials and Methods

### Cells, Plasmids and Reagents

SKBR-3, HeLa and A431 cells were obtained from the American Type Culture Collection (ATCC, Manassas, VA) and grown according to their specifications. F1-4, a CHO subline stably expressing ErbB1-EGFP has been described previously^51^. Information about which cell line was used for the different experiments is available in Supplementary Materials and Methods. For experiments involving growth factor stimulation serum-starved cells were incubated with 100 ng/ml (~16 nM) of recombinant human EGF (R&D Systems, Minneapolis, MN or Peprotech, London, UK) for 5 min at 37 °C in Tyrode’s buffer with 10 mM glucose and 0.1% BSA. The pSV2neuNT plasmid coding for the human NeuT protein was a kind gift from Richard Pestell (Thomas Jefferson University, Philadelphia, PA)^52^. Cloning of mYFP-tagged Val659Glu mutant of ErbB2 is described in Supplementary Materials and Methods.

### Labeling of antibodies and cells

For microscopic experiments, cells were cultured on Lab-Tek II chambered coverglass (Nalge Nunc International, Rochester, NY). For flow cytometry, cells were harvested by trypsinization. Trastuzumab (against ErbB2) and Ab11 (against ErbB1) were purchased from Roche (Basel, Switzerland) and Thermo Fisher Scientific (Waltham, MA), respectively. These antibodies were labeled with AlexaFluor546 and AlexaFluor647 dyes according to the manufacturer’s specifications (Life Technologies/Thermo Fisher Scientific, Waltham, MA). Antibodies against phosphotyrosine (PY99, Santa Cruz, Dallas, TX), ErbB2 phosphorylated at Tyr1248 (Ab18, Thermo Fisher Scientific) and ErbB1 phosphorylated at Tyr1068 (1H12, Cell Signaling Technology, Danvers, MA) were visualized by secondary staining with AlexaFluor546-goat-anti-mouse antibodies (Life Technologies/Thermo Fisher Scientific). Tetramethylrhodamine-labeled EGF was purchased from LifeTechnologies. Transient transfection of HeLa cells was carried out with an Amaxa Nucleofector device (Lonza, Basel, Switzerland). The transfection solution and the program were selected according to the “Cell & Transfection Database” of the manufacturer.

### Measuring and Altering the Dipole Potential

The dipole potential was measured using a ratiometric assay. Cells were incubated with 2 μM di-8-ANEPPS (4-(2-[6-(dioctylamino)-2-naphthalenyl]ethenyl)-1-(3-sulfopropyl)pyridinium inner salt, Invitrogen, Carlsbad, CA) for 20 min at 4 °C, and their fluorescence was measured using a ratiometric assay shown to be responsive to changes in the dipole potential^53^. The dye was excited at 458 and 514 nm, and its emission was measured above 630 nm in order to avoid fluidity-induced changes in the excitation ratio^54^. Image acquisition was performed using an LSM510 confocal laser scanning microscope (Carl Zeiss AG, Jena, Germany). Image processing was carried out with the DipImage toolbox (Delft University of Technology, Delft, The Netherlands) under Matlab (Mathworks, Natick, MA). Segmentation of images into membrane and non-membrane pixels was carried out with the manually seeded watershed algorithm using a custom-written Matlab program as described previously^55^. The fluorescence intensity ratio of the cell membrane pixels was calculated after background subtraction. The dipole potential was increased and decreased by treating cells with 6-ketocholestanol (3β-hydroxy-5α-cholestan-6-one) (Sigma Aldrich, St. Louis, MO) and phloretin (3-(4-hydroxyphenyl)-1-(2,4,6-trihydroxyphenyl)propan-1-one) (Sigma Aldrich, St. Louis, MO), respectively at a concentration of 100 μM for 10 min at room temperature in the presence of 0.05% (v/v) Pluronic F-127^54^.

### Determination of the correlation between tyrosine phosphorylation of ErbB2, its raft localization and the dipole potential

Control cells and those with an altered dipole potential were treated with EGF followed by staining with subunit B of cholera toxin (CTX) and antibodies against ErbB2 and tyrosine phosphorylated ErbB2. Details of the labeling protocol and image analysis are described in Supplementary Materials and Methods.

### Determination of the binding affinity of EGF

The dissociation constant characterizing the binding affinity of EGF to ErbB1 was determined by competitive and non-competitive binding assays using fluorescently labeled EGF. Tetramethylrhodamine-congjutated EGF (TAMRA-EGF, LifeTechnologies) was reconstituted at a concentration of 10 μM. A dilution series was prepared with each vial containing 250 μl EGF diluted in Tyrode’s buffer supplemented with 0.1% (w/v) BSA. The solutions were kept on ice before adding 20 μl of cold cell suspension containing 100000 cells. Cells were incubated in the presence of TAMRA-EGF for 30 min on ice with shaking. The fluorescence intensity of the samples was measured on a FACS Aria III flow cytometer without washing to prevent the dissociation of fluorescent EGF from ErbB1. TAMRA was excited at 561 nm, and its emission was detected through a 595 nm band-pass filter. Analysis of flow cytometric data was carried out with FCS Express (Denovo Software, Thornhill, Ontario, Canada). The binding curves were fitted to the Hill equation on a logarithmic scale:


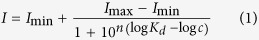


where *I* is the intensity of the sample labeled with fluorescent EGF at a concentration of *c, I*_min_ and *I*_max_ are the minimal and maximal intensities, respectively. *K*_d_ and *n* are the dissociation constant and the Hill coefficient, respectively.

Competitive binding curves were measured as described above, but the dilution series contained unlabeled EGF mixed with a constant concentration of labeled EGF. The binding curves were fitted to the equation below:


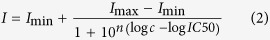


where


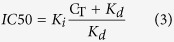


*K*_d_ and *K*_i_ are the dissociation constants of TAMRA-EGF and unlabeled EGF, respectively, and c_T_ is the concentration of TAMRA-EGF.

### Flow Cytometric Förster Resonance Energy Transfer (FRET) Measurements

ErbB1 homoassociation and ErbB2 homoassociation were measured with Ab11 antibody and trastuzumab, respectively. ErbB1-ErbB2 heteroassociation was measured with AlexaFluor546-Ab11 (against ErbB1) and AlexaFluor647-trastuzumab (against ErbB2). The measurements were carried out with a FACS Aria III flow cytometer (BD Biosciences, San Jose, CA) according to principles described in Supplementary Materials and Methods.

### Number & Brightness Analysis (N&B)

An Olympus FV1000 confocal microscope running in pseudo photon-counting mode was used to carry out N&B analysis according to Digman *et al*.^56^. Cells were kept in Tyrode’s buffer with 10 mM glucose and 0.1% BSA during the measurements. mYFP and GFP were excited at 514 nm and 488 nm, respectively, and their emission was recorded between 530–630 nm and 500–600 nm, respectively. Image series of 100 optical slices adjacent to the coverslip were recorded from live cells to determine the variance of the fluorescence intensity. See Supplementary Materials and Methods for additional details of the measurement.

## Additional Information

**How to cite this article**: Kovács, T. *et al*. The Dipole Potential Modifies the Clustering and Ligand Binding Affinity of ErbB Proteins and Their Signaling Efficiency. *Sci. Rep.* **6**, 35850; doi: 10.1038/srep35850 (2016).

## Supplementary Material

Supplementary Information

## Figures and Tables

**Table 1:** Binding affinity of fluorescent and unlabeled EGF to ErbB1 at different dipole potentials.

Treatment	SKBR-3			HeLa			A431		
	non-competitive	competitive		non-competitive	competitive		non-competitive	competitive	
	*K*_d_ (labeled) [nM]	IC50 [nM]	*K*_i_ [nM]	*K*_d_ (labeled) [nM]	IC50 [nM]	*K*_*i*_[nM]	*K*_d_ (labeled) [nM]	IC50 [nM]	*K*_i_ [nM]
Pluronic F-127	0.37 ± 0.07	6.44 ± 0.94	0.44 ± 0.1	2.1 ± 0.12	13.9 ± 1.65	4.1 ± 0.31	0.99 ± 0.1	6.1 ± 0.8	1 ± 0.15
phloretin	0.29 ± 0.05	5.3 ± 0.5	0.3 ± 0.07	2.47 ± 0.2	11.8 ± 1.9	3.9 ± 0.27	0.98 ± 0.08	7 ± 0.7	1.15 ± 0.18
6-ketocholestanol	7.23* ± 0.37	15.9* ± 1.3	9.4* ± 1.02	18.6* ± 0.25	33.5* ± 3.9	26.4* ± 1.62	1.63* ± 0.21	10.6* ± 1	2.6* ± 0.33

Control cells (Pluronic F-127) or those with an altered dipole potential (phloretin, 6-ketocholestanol) were incubated with a dilution series of fluorescent EGF and the *K*_d_ was determined by fitting the Hill equation to the measured data points (non-competitive). Alternatively, cells were incubated with a dilution series of unlabeled EGF in the presence of a constant concentration of fluorescent EGF. The *IC50* was determined by fitting and the *K*_i_ values were calculated using the *K*_d_ values determined previously. The original curves are shown in Fig. S2 (*p < 0.05, compared to Pluronic F-127-treated control cells by ANOVA followed by Tukey’s HSD test).
