# Supplementary material for: The Dipole Potential Modifies the Clustering and Ligand Binding Affinity of ErbB Proteins and Their Signaling Efficiency
Source: Sci Rep. 2016 Oct 24;6:35850. doi: 10.1038/srep35850 (PMC5075772; doi:10.1038/srep35850)
Supplement: Supplementary Information [file srep35850-s1.pdf]

Supplementary material for

The Dipole Potential Modifies the Clustering and Ligand Binding Affinity of ErbB Proteins and Their Signaling Efficiency

Tamás Kovács, Gyula Batta, Tímea Hajdu, Ágnes Szabó, Tímea Váradi, Florina Zákány, István Csomós, János Szöllősi, Peter Nagy

Supplementary Materials and Methods

Cells

The table below shows the cells types used for carrying out FRET and N&B experiments:

	FRET	N&B
ErbB1 homoassociation	HeLa	F1-4
ErbB2 homoassociation	SKBR-3	HeLa (transient transfection)
ErbB1-2 heteroassociation	SKBR-3	-
NeuT homoassociation	HeLa (transient transfection)	HeLa (transient transfection)
homoassociation of transfected wild-type ErbB2	HeLa (transient transfection)	-

The cell lines have been characterized with respect to the number of ErbB receptors/cell by Qifikit (Dako, Glostrup, Denmark) as shown in the following table:

Cell line	Number of receptors/cell ($\cdot 10^3$)	
	ErbB1	ErbB2
A431	2300	25
HeLa	50	31
F1-4	610 (ErbB1-GFP)	0
SKBR-3	190	1100

HeLa and SKBR-3 were selected for the flow cytometric FRET measurement of the homoassociation of ErbB1 and ErbB2, respectively, because they express high enough numbers of these receptors. Due to the extremely high expression of ErbB1 A431 cells must contain preformed ErbB1 dimers making detection of EGF-induced effects on ErbB1 clustering more difficult[1]. Therefore, A431 cells were only used for measuring equilibrium binding of EGF. SKBR-3 cells were selected for the measurement of ErbB1-2 heteroassociation since this cell line expresses relatively high levels of both receptors. N&B analysis of the homoassociation of ErbB1 was performed in F1-4 cells since they stably express a high number of ErbB1-GFP. Since a cell line stably expressing a fluorescent protein-conjugated version of ErbB2 was not available, HeLa cells were transiently transfected with ErbB2-mYFP for the measurement of ErbB2 homoassociation by N&B analysis.

Cloning of mYFP-tagged ErbB2 harboring a Val→Glu mutation in the transmembrane domain

The mYFP tagged, Val659Glu mutant human ErbB2 (NeuT) was generated by introducing a single amino acid substitution into PCDNA3.1 ErbB2-short-mYFP expressing wild-type ErbB2 fused to mYFP[1] using the NEB Q5® Site-Directed Mutagenesis Kit (NEB, E0554S). The forward (TCTGCGGTG<u>GAA</u>GGCATTCTGC) and reverse primers (GATGATGGACGTCAGAGG) applied for the amplification of the plasmid and the mutagenesis were designed with the NEBaseChanger™ online application. The presence of the desired mutation was confirmed by sequencing. The plasmid was named PCDNA3.1 ErbB2 (Val659Glu)-shortmYFP.

Functional Analysis of the Effect of the Dipole Potential Using Flow Cytometry

Cells fixed in 3.7% formaldehyde for 30 min on ice were labeled with antibodies against phosphotyrosine or tyrosine-phosphorylated ErbB1 or ErbB2 in PBS containing 0.1% BSA and 0.1% Triton X-100 followed by washing and secondary labeling with AlexaFluor546-goat-anti-mouse antibody. Both primary and secondary labeling was carried out for 30 min on ice. Fluorescence intensities measured with a FACS Aria III flow cytometer (BD Biosciences, San Jose, CA) were analyzed on a cell-by-cell basis.

Determination of the correlation between tyrosine phosphorylation of ErbB2, its raft localization and the dipole potential

Cells were serum-starved overnight followed by treatment with 6-ketocholestanol to increase their dipole potential. Control cells were only treated with Pluronic F127. Afterwards cells were stimulated with 100 ng/ml (≈16 nM) EGF for 5 min at 37°C in Tyrode's buffer with 10 mM glucose and 0.1% BSA followed by staining with AlexaFluor546-trastuzumab and AlexaFluor647-CTX-B (subunit B of cholera toxin) on ice. CTX-B binds to GM1 gangliosides known to be concentrated in lipid rafts. Therefore, CTX-B labeling is often used as a lipid raft marker. Afterwards cells were fixed in 3.7% formaldehyde and labeled with Ab18 (against phosphorylated ErbB2) followed by secondary staining with AlexaFluor488-GAMIG (to visualize Ab18 binding). Labeling steps after the formaldehyde fixation were performed in PBS containing 0.1% BSA and 0.1% Triton X-100.

Cells were manually identified in images followed by segmentation into two masks corresponding to high and low CTX intensities. The intensity of Ab18 staining (corresponding

to tyrosine phosphorylated ErbB2) normalized to the expression level of ErbB2 (trastuzumab staining) was separately calculated for the high-CTX and low-CTX masks corresponding to raft and non-raft domains, respectively. The Pearson correlation coefficient was calculated between the Ab18 intensity and CTX staining and between trastuzumab intensity and CTX staining. Image analysis was carried out in Matlab.

Flow Cytometric Förster Resonance Energy Transfer (FRET) Measurements

AlexaFluor546- and AlexaFluor647-conjugated antibodies were used as donor and acceptor, respectively. The donor, measured in the donor channel, was excited at 561 nm and its emission was detected between 570-620 nm. The fluorescence of AlexaFluor647, measured in the acceptor channel, was excited at 633 nm and detected with a 635LP filter. Besides the donor (I_1) and acceptor (I_3) channels the fluorescence intensity of cells was also measured in the FRET channel (I_2) corresponding to the donor excitation and acceptor emission wavelengths. Spectroscopic overspill parameters were determined using cells labeled with only donor- or acceptor-conjugated antibodies. Measurements were carried out with a FACS Aria III flow cytometer (BD Biosciences, San Jose, CA). The FRET efficiency was evaluated on a cell-by-cell basis using the ReFlex software by solving the following equation set for E, the FRET efficiency[2,3]:

$$\begin{aligned} I_1 &= I_D(1-E) + I_A S_4 + I_D\, E\, \alpha \frac{S_4}{S_2} \\ I_2 &= I_D(1-E) S_1 + I_A S_2 + I_D\, E\, \alpha \\ I_3 &= I_D(1-E) S_3 + I_A + I_D\, E\, \frac{\varepsilon_{R2}}{S_2},\ \varepsilon_{R2} = \frac{\varepsilon_D{}^A\ \varepsilon_A{}^D}{\varepsilon_D{}^D\ \varepsilon_A{}^A} \end{aligned} \tag{1}$$

where S_1 and S_3 characterize the overspill of donor emission to the FRET and acceptor channels, respectively, and S_2 and S_4 describe the overspill of acceptor emission to the FRET and donor channels, respectively. I_D and I_A are the unquenched donor intensity and the directly excited acceptor intensity, respectively. α relates the intensity of an excited acceptor molecule detected in the FRET channel to that of an excited donor molecule detected in the donor channel according to the following equation:

$$\alpha = \frac{Q_A}{Q_D} \frac{\eta_{A,2}}{\eta_{D,1}} \tag{2}$$

where Q_A and Q_D are the fluorescence quantum efficiencies of the acceptor and donor, respectively, and $\eta_{A,2}$ and $\eta_{D,1}$ characterize the detection efficiency of an acceptor photon in the FRET channel and that of a donor photon in the donor channel, respectively. ε stands for the molar absorption coefficient of the donor or acceptor indicated in the subscript measured at the emission range of the species labeled in the superscript.

The homoclustering of ErbB1 was measured in cells labeled by a mixture of AlexaFluor546-Ab11 and AlexaFluor647-Ab11, whereas the homoclustering of ErbB2 was analyzed in cells labeled by a mixture of AlexaFluor546-trastuzumab and AlexaFluor647-trastuzumab. ErbB1-ErbB2 heteroassociation was measured with AlexaFluor546-Ab11 and AlexaFluor647-trastuzumab used as the donor and acceptor, respectively.

Number & Brightness Analysis (N&B)

GFP and mYFP were excited at 488 nm and 514 nm, respectively, and their emission was recorded between 500-600 nm and 530-630 nm, respectively. N&B experiments were performed with an Olympus FV1000 confocal microscope running in pseudo photon-counting mode according to Digman et al[4]. Cells were kept in Tyrode's buffer with 10 mM glucose and 0.1% BSA during the measurements. Image series of 100 optical slices adjacent to the coverslip were recorded from live cells to determine the variance of the fluorescence intensity with a pixel size of 82 nm and pixel dwell time of 10 µs. A single image consisted of 512 × 512 pixels and the central part of images with pixels corresponding to the membrane was used for analysis to eliminate artifacts arising from scanner speed nonlinearity at the borders. The image stack was analyzed with a custom-written Matlab program (NB_tools) incorporating functions of the DipImage toolbox[1]. The images were first registered (i.e., corrected for lateral shift) followed by calculating the mean and variance of every pixel. The apparent brightness was calculated according to the following equation:

$$B = \frac{\sigma^2}{\langle k \rangle} = \varepsilon + 1 \tag{3}$$

where σ^2 and $\langle k \rangle$ are the variance and the mean, respectively, of a given pixel. The molecular brightness (ε) characterizes the clustering state of a fluorescent molecule by giving the number of photons detected from a single diffusing unit during the pixel dwell time. If the image mean decreased by more than 10% due to stage shift or photobleaching or if the pixel variance did not converge to zero with increasing stack size, the stack was discarded.

Supplementary Results

Phloretin and 6-ketocholestanol Alter the Dipole Potential

It has been shown that the dipole potential can be increased by incorporating 6-ketocholestanol into the membrane, whereas phloretin decreases the dipole potential[5]. Labeling with the ratiometric dye, di-8-ANEPPS, was carried out either before or after treating the cells with the agents altering the dipole potential. The experiments showed that 6-ketocholestanol increased, whereas phloretin decreased the dipole potential of the plasma membrane in all cell types used in the current paper. The fact whether labeling with the indicator preceded or followed the treatment with the agents altering the dipole potential did not modify the findings. Representative results for SKBR-3 cells are presented in Fig. S1. Based on fluorescence microscopy the accumulation of the fluorescent indicator was restricted to the plasma membrane. Therefore, we concluded that 6-ketocholetanol and phloretin can modify the dipole potential of the plasma membrane and proceeded to investigate the effect of these alterations on the clustering and signaling properties of ErbB plasma membrane receptors.

Supplementary Figures

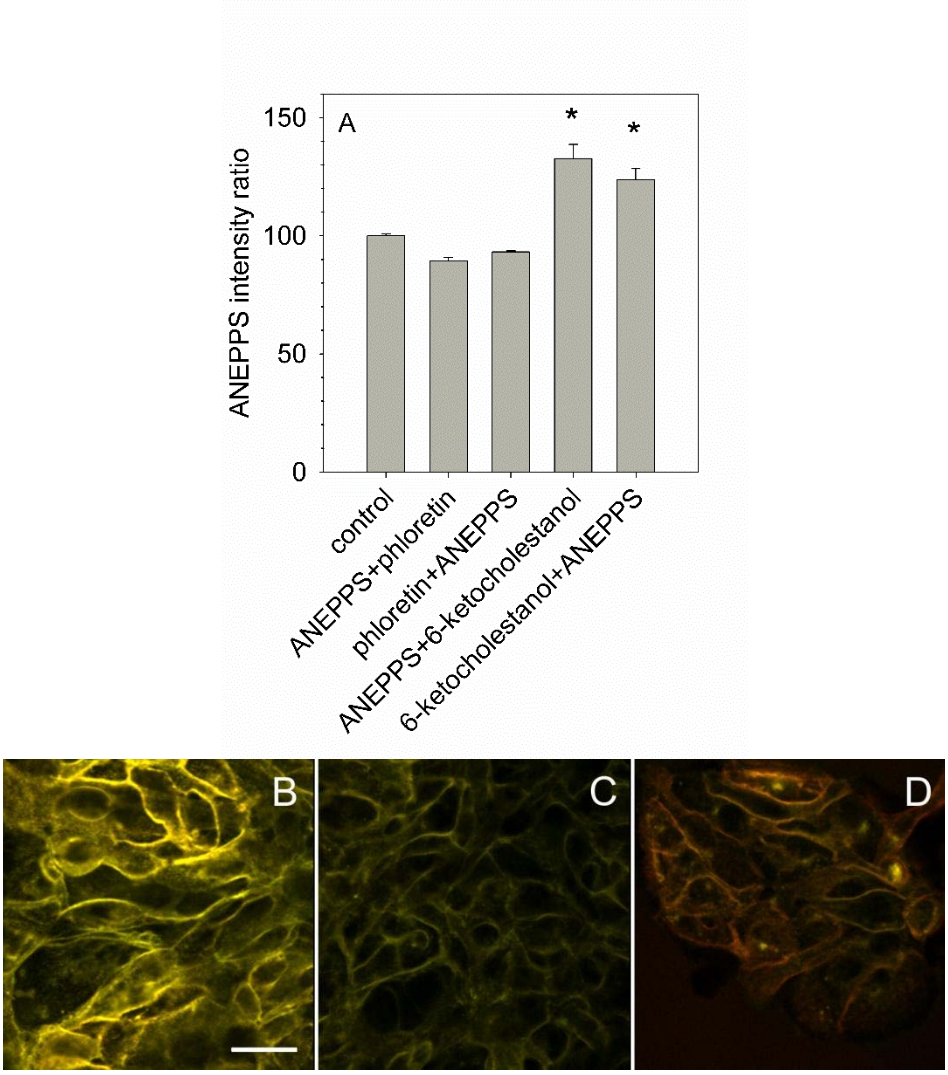

Fig. S1. Ratiometric detection of the alteration of the dipole potential. A. Quantitative evaluation of the dipole potential. SKBR-3 cells were treated with phloretin and 6-ketocholestanol to decrease and increase the dipole potential, respectively. Samples labeled with “ANEPPS+ketocholestanol” and “ANEPPS+phloretin” were stained with ANEPPS before 6-ketocholestanol and phloretin treatment, while in the case of the other two treated samples staining with ANEPPS was performed after the treatments. The fluorescence intensity ratios for the treated and the control cells were normalized to the sample treated with Pluronic F127 (“control”). Asterisks indicate significant differences compared to samples treated with Pluronic F127 ($p < 0.05$, ANOVA followed by Tukey’s HSD test). B-D. Representative images of control (B), phloretin (C) and 6-ketocholestanol (D)-treated cells. The red and green channels in the composite color images correspond to the fluorescence detected by excitation at 458 nm and 514 nm, respectively. The scale bar, valid for every image, is 20 µm.

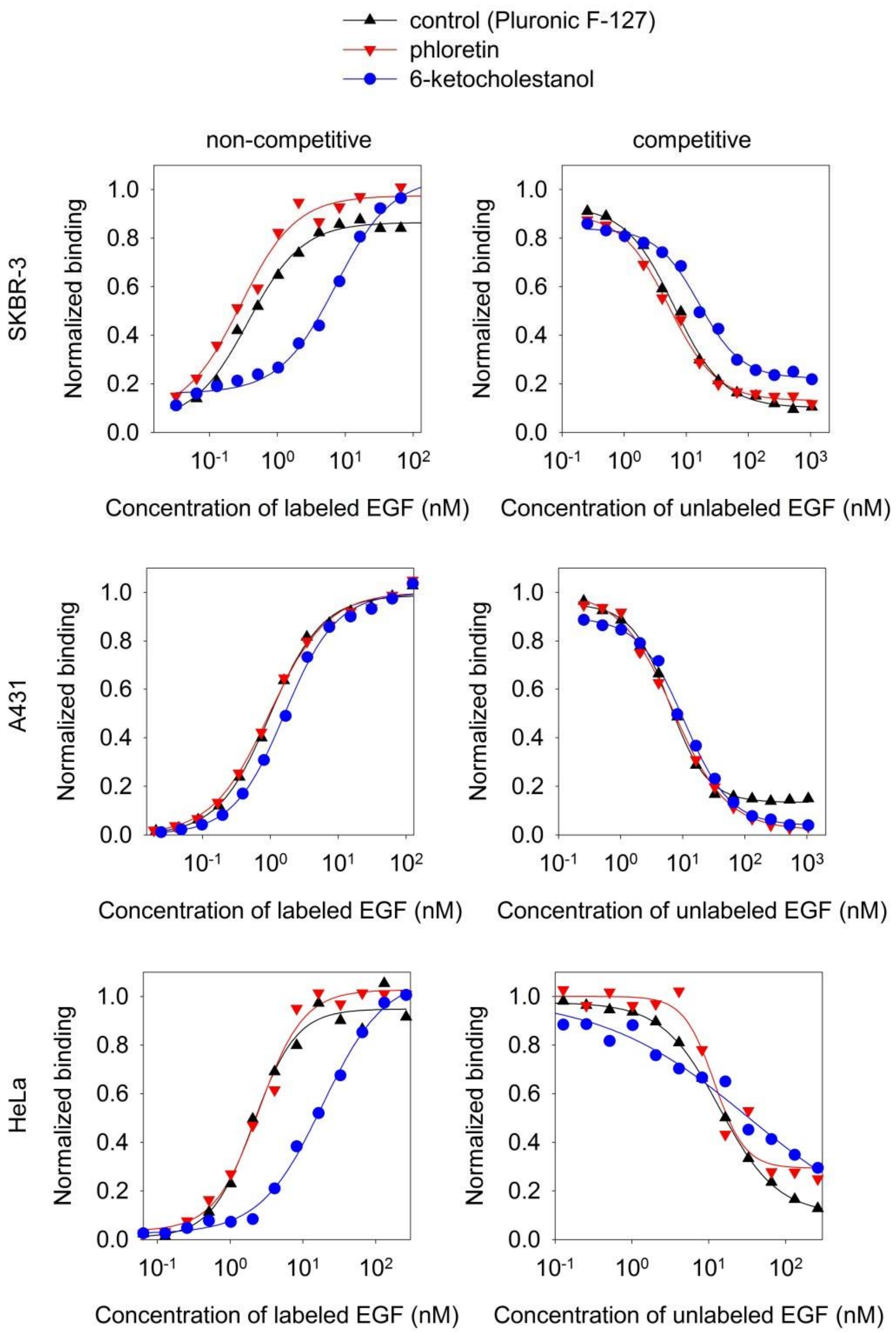

Figure S2. Effect of the dipole potential on the binding affinity of EGF. Control cells and those with increased (6-ketocholestanol) and decreased (phloretin) dipole potential were labeled with a dilution series of TAMRA-EGF (non-competitive) or with a dilution series of unlabeled EGF in the presence of 5 nM of TAMRA-EGF (competitive). The fluorescence intensity of each sample measured by flow cytometry is plotted in a semi-logarithmic plot (symbols). The lines are fits of the Hill equation to the measured data. The derived K_d and K_i values are shown in Table 1.

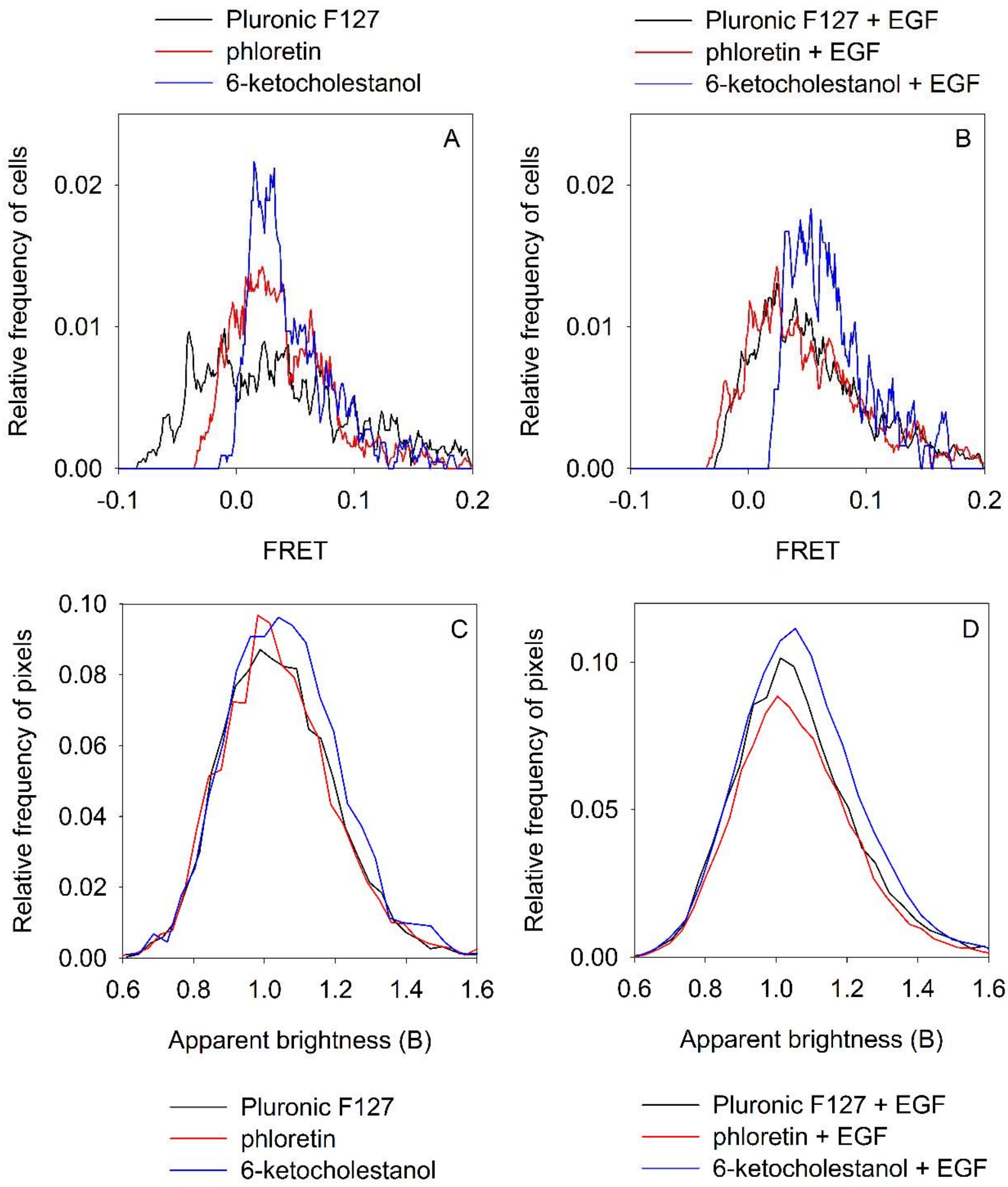

Figure S3. Representative figures of FRET and N&B measurements. A-B. The homoassociation of ErbB1 was measured by flow cytometric FRET under the conditions indicated in the legends in the presence or absence of EGF stimulation. C-D. The homoassociation of ErbB2 was analyzed by N&B measurements under the conditions indicated in the legends in the presence and absence of EGF stimulation.

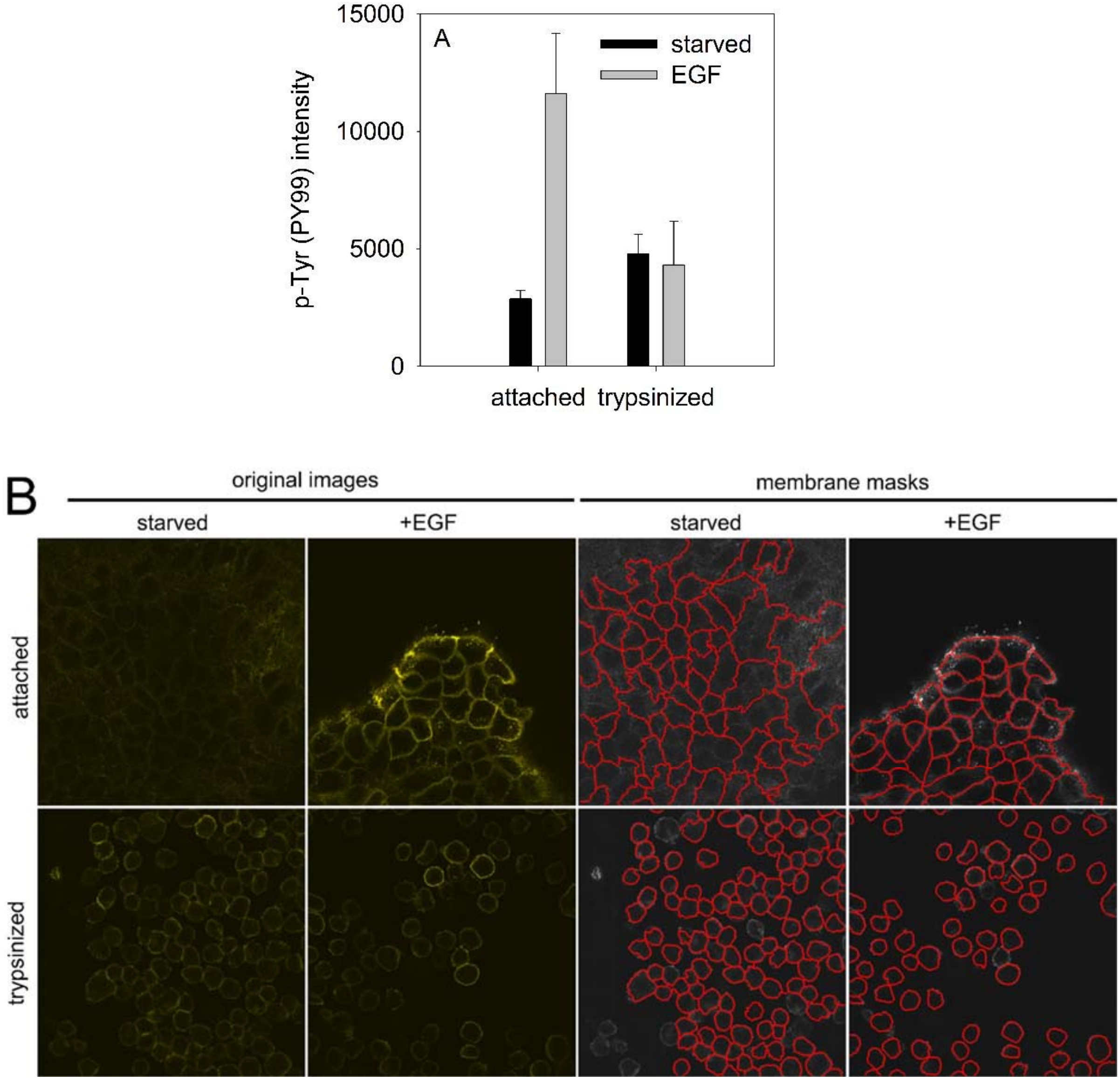

Fig. S4. Effect of trypsinization on the activation state of cells. Starved SKBR-3 cells were either stimulated with EGF without trypsinization or stimulation was carried out in suspended cells after trypsinization. Fixed and permeabilized cells were stained with PY99 against p-Tyr followed by secondary staining. The fluorescence intensity was evaluated in the membrane identified by manually-seeded watershed segmentation. The average fluorescence intensities (±standard error of the mean determined from three independent measurements) are plotted in A and representative original images alongside the membrane masks are displayed in B.

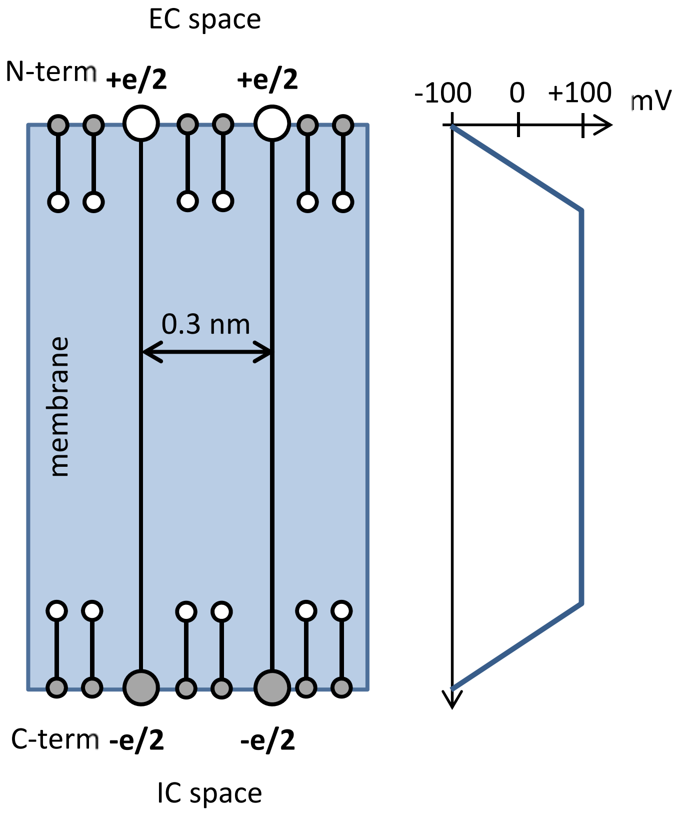

Figure S5. Interaction with the net dipole of the transmembrane domains with the membrane dipole potential. The net dipole of transmembrane α-helices is approximated by placing half elementary charges at the N- and C-termini of the helix. The membrane dipole potential is assumed to arise from the ordered alignment of dipoles near the surface of the membrane. Since the dipole potential is positive inside the membrane, negative charges, designated by gray circles, must be present on the membrane surface, while positive charges, denoted by white circles, are located deeper in the membrane. The magnitude and profile of the membrane dipole potential is in accordance with Flewelling[6]. The dielectric constant (ε_r, relative permittivity) of the lipid headgroup region is assumed to be 10 (Ref [7]). Under these conditions the electric potential generated by the positive half unit charge at the N-terminus at a distance of r=0.3 nm, i.e. at approximately the other transmembrane domain is

$$U = \frac{1}{4\pi\varepsilon_0\varepsilon_r}\frac{0.5e}{r} = 8.9\cdot 10^8 \frac{0.5\cdot 1.6\cdot 10^{-19}}{0.3\cdot 10^{-9}} = 237\text{ mV} \qquad (4)$$

Although the calculation is simple, the magnitude of this potential is on the order of the membrane dipole potential. Since the sign of the membrane dipole potential at the N-terminus is opposite to this potential, it will counterbalance the repulsion of the α helices. On

the other hand, the field generated by the dipole moment of the α helices and the membrane dipole potential will strengthen each other at the C-terminus. Consequently, the membrane dipole potential, by counterbalancing the electrostatic repulsion between the α helical transmembrane domains at the N-terminus, favors the formation of active dimers stabilized by the N-terminal dimerization motifs, while it has no such effect on inactive dimers stabilized by the C-terminal dimerization motifs.

Supplementary References

1 Nagy, P., Claus, J., Jovin, T. M. & Arndt-Jovin, D. J. Distribution of resting and ligand-bound ErbB1 and ErbB2 receptor tyrosine kinases in living cells using number and brightness analysis. *Proc Natl Acad Sci U S A* **107**, 16524-16529 (2010).

2 Nagy, P., Vereb, G., Damjanovich, S., Mátyus, L. & Szöllősi, J. in *Current Protocols in Cytometry* (ed J.P. Robinson) Ch. 12.8, 12.18.11-12.18.13 (John Wiley & Sons, 2006).

3 Szentesi, G. *et al.* Computer program for determining fluorescence resonance energy transfer efficiency from flow cytometric data on a cell-by-cell basis. *Comput Methods Programs Biomed* **75**, 201-211 (2004).

4 Digman, M. A., Dalal, R., Horwitz, A. F. & Gratton, E. Mapping the number of molecules and brightness in the laser scanning microscope. *Biophys J* **94**, 2320-2332 (2008).

5 Gross, E., Bedlack, R. S., Jr. & Loew, L. M. Dual-wavelength ratiometric fluorescence measurement of the membrane dipole potential. *Biophys J* **67**, 208-216 (1994).

6 Flewelling, R. F. & Hubbell, W. L. The membrane dipole potential in a total membrane potential model. Applications to hydrophobic ion interactions with membranes. *Biophys J* **49**, 541-552 (1986).

7 Sengupta, D., Meinhold, L., Langosch, D., Ullmann, G. M. & Smith, J. C. Understanding the energetics of helical peptide orientation in membranes. *Proteins* **58**, 913-922 (2005).
